# CD73's Potential as an Immunotherapy Target in Gastrointestinal Cancers

**DOI:** 10.3389/fimmu.2020.00508

**Published:** 2020-04-15

**Authors:** Jerry B. Harvey, Luan H. Phan, Oscar E. Villarreal, Jessica L. Bowser

**Affiliations:** ^1^Department of Anesthesiology, The University of Texas Health Science Center at Houston, Houston, TX, United States; ^2^Department of Gastrointestinal Medical Oncology, The University of Texas MD Anderson Cancer Center, Houston, TX, United States

**Keywords:** CD73, adenosine, gastrointestinal cancers, immunosuppression, immunotherapy

## Abstract

CD73, a cell surface 5′nucleotidase that generates adenosine, has emerged as an attractive therapeutic target for reprogramming cancer cells and the tumor microenvironment to dampen antitumor immune cell evasion. Decades of studies have paved the way for these findings, starting with the discovery of adenosine signaling, particularly adenosine A2A receptor (A2AR) signaling, as a potent suppressor of tissue-devastating immune cell responses, and evolving with studies focusing on CD73 in breast cancer, melanoma, and non-small cell lung cancer. Gastrointestinal (GI) cancers are a major cause of cancer-related deaths. Evidence is mounting that shows promise for improving patient outcomes through incorporation of immunomodulatory strategies as single agents or in combination with current treatment options. Recently, several immune checkpoint inhibitors received FDA approval for use in GI cancers; however, clinical benefit is limited. Investigating molecular mechanisms promoting immunosuppression, such as CD73, in GI cancers can aid in current efforts to extend the efficacy of immunotherapy to more patients. In this review, we discuss current clinical and basic research studies on CD73 in GI cancers, including gastric, liver, pancreatic, and colorectal cancer, with special focus on the potential of CD73 as an immunotherapy target in these cancers. We also present a summary of current clinical studies targeting CD73 and/or A2AR and combination of these therapies with immune checkpoint inhibitors.

## Introduction

Gastrointestinal (GI) cancers are some of the most common cancers worldwide and a major cause of cancer-related deaths (1–5). Immune checkpoint inhibitors (ICIs), including pembrolizumab (Keytruda) and nivolumab (Opdivo), antibodies against programmed death-1 (PD-1), recently gained Food and Drug Administration (FDA) approval for use in GI cancers (Table 1) (6–11). While their approval has been a significant step forward in advancing clinical care, currently, few patients benefit (12). Patients benefiting the most tend to have tumors harboring deficient DNA mismatch repair (dMMR) and high microsatellite instability (MSI-H) (8, 13). dMMR and MSI-H occur together at a consistency of 90–95% (referred to as dMMR/MSI-H) (14, 15). MMR deficiency leads to high mutational rates and subsequently high presence of neoantigens, making tumor cells more likely to be recognized and destroyed by antitumor immune cells (8, 13, 16–18). Tumor-infiltrating lymphocytes are abundant in dMMR/MSI-H tumors and associate with favorable prognosis (19, 20). For comparison, the somatic mutation frequency of dMMR/MSI-H tumors is 10–100-fold to that of proficient MMR tumors (21). In contrast, ICIs as single agents have not shown meaningful benefit for proficient MMR tumors (8), which are the vast majority of GI cancer cases. MSI-H tumors account for 6–22% of gastric, 1% of pancreatic, 3% of liver, and 14–16% of colorectal cancers (22–27). Antibodies against PD-1/programmed death-ligand 1 (PD-L1) or cytotoxic T-lymphocyte-associated protein-4 (CTLA-4) are the most clinically advanced immunotherapy in cancer (12). The PD-1/PD-L1 axis promotes adaptive immune resistance by suppressing effector T cells and promoting the differentiation of regulatory T cells (Tregs). CTLA-4 also is a negative regulator of T cells; its engagement of B7-1 or B7-2 on antigen-presenting cells inhibits T cell activation (12). Preclinical and clinical efforts are pushing forward with combination ICI therapy as well as ushering in different approaches to harness the immune system to extend immunotherapy efficacy to more patients, including vaccines and viral therapy, adoptive cell transfer, and cytokine treatment (12, 28). Challenges with improving efficacy include overcoming immunosuppression activity by the tumor microenvironment, unmasking pre-existing immune cell activity, and the ability to stimulate *de novo* immunogenicity (29). In recent years, antibodies and small molecular inhibitors against CD73 have made their way into clinical trials as an attractive target for restoring antitumor immunity (30–44). This review provides a summary of current literature for CD73 in GI cancers and its potential as an immunotherapy target. We also discuss current clinical trials targeting CD73 and adenosine receptors in combination with ICI and conventional therapy and the clinical implications to GI tumors.

**Table 1:** Summary of Food and Drug Administration approved immune checkpoint inhibitors in GI cancers.

**Drug(s)**	**Target(s)**	**Therapy modality**	**Tumor type**	**Details**	**Objective response rate (%)**	**FDA approved Year**	**Clinical trial**	**ClinicalTrials.gov identifier**	**References (PMID)**
Pembrolizumab (Keytruda)	PD-1	Humanized monocolonal antibody	Gastric Cancer	Patients with recurrent locally advanced or metastatic gastric or gastroesophageal junction adenocarcinoma whose tumors express PD-L1	60.0% (combination with cisplatin) 25.8% (single agent)	2017	KEYNOTE-059	NCT02335411	30911859
			Liver Cancer	Patients with hepatocellular carcinoma who previously received sorafenib	17%	2018	KEYNOTE-224	NCT02702414	29875066
			Colorectal Cancer	Patients with microsatellite instability-high (MSI-H) or deficient mismatch repair (dMMR) unresectable or metastatic colorectal cancer that has progressed following treatment with fluoropyrimidine, oxaliplatin, and irinotecan Also approved for any solid tumor that has tested positive for MSI-H or dMMR in patients who have had prior treatment and have no satisfactory alternative treatment options	Colorectal Cancer: 40% (dMMR) 0% (proficient MMR) Non-colorectal Cancers: 70% (dMMR)	2017	KEYNOTE	NCT01876511	26028255
Nivolumab (Opdivo)	PD-1	Humanized monocolonal antibody	Liver Cancer	Patients with advanced hepatocellular carcinoma. The approval covers the use of nivolumab in patients who have previously received sorafenib	15, 20%	2017	CheckMate 040	NCT01658878	28434648
			Colorectal Cancer	Patients with MSI-H or dMMR metastatic colorectal cancer that has progressed following treatment with fluoropyrimidine, oxaliplatin, and irinotecan	68.9%	2017	CheckMate 142	NCT02060188	28734759
Nivolumab (Opdivo) Ipilimumab (Yervoy)	PD-1 CTLA-4	Humanized monocolonal antibodies	Colorectal Cancer	Patients with MSI-H or dMMR metastatic colorectal cancer that has progressed following treatment with fluoropyrimidine, oxaliplatin, and irinotecan.	55%	2018	CheckMate 142	NCT02060188	29355075

## CD73 and Adenosine Receptor Activity Promotes Immunosuppression

**Figure 1:**
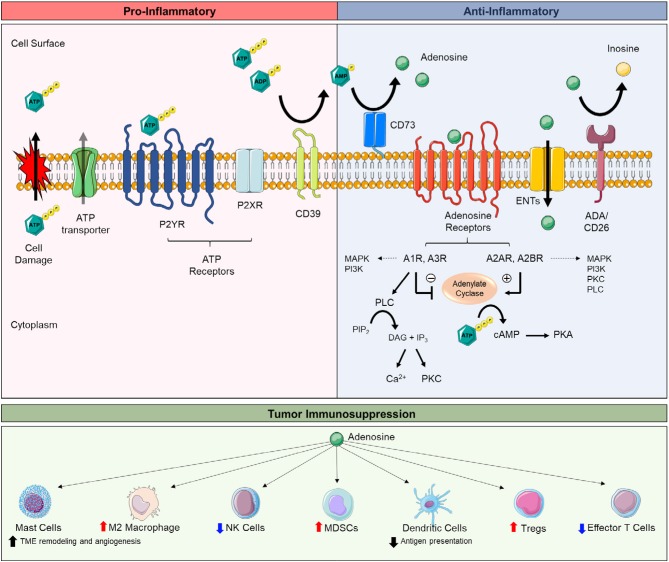
Extracellular adenosine synthesis, adenosine receptor signaling, and adenosine-mediated immunosuppression. Extracellular adenosine and receptor signaling is part of a large cascade of ecto-enzymes (e.g., CD39, CD73), membrane transporters (e.g., ENTs), and G-protein-coupled (e.g., P2YR, adenosine receptors) and ionotrophic receptors (e.g., P2XR) known as the purinergic pathway. The purinergic pathway mediates both pro-inflammatory and anti-inflammatory responses. The breakdown of extracellular adenosine triphosphate (ATP) to extracellular adenosine is key to balancing tissue inflammation. Intracellular ATP is released by lytic (e.g., stressed and/or apoptotic/necrotic cells) and non-lytic (e.g., pannexin-1 and connexins) routes secondary to tissue damage, inflammation, and/or hypoxia. Once released, ATP activates ATP receptors (e.g., P2XR and P2YR) to promote pro-inflammatory responses, including the release of inflammatory cytokines promote lymphocyte proliferation, cell mobility, and phagocyte recruitment. ATP is dephosphorylated to extracellular adenosine by CD39, converting ATP and adenosine diphosphate (ADP) to adenosine monophosphate (AMP), and CD73, converting AMP to adenosine. Extracellular adenosine signaling through adenosine receptors (e.g., A1R, A2AR, A2BR, A3R) promotes anti-inflammatory responses, including the release of pro-tolerance cytokines, regulatory lymphocytes, and skewing toward M2 macrophages. Extracellular adenosine also can be taken up intracellularly by equilibrative nucleoside transporters (e.g., ENTs) or be further metabolized to inosine (e.g., ADA/CD26). A2AR and A2BR signaling stimulate adenylate cyclase to produce cyclic AMP (cAMP) which activates protein kinase A (PKA). A1R and A3R signaling inhibit adenylate cyclase. Adenosine receptors can activate multiple signaling pathways (e.g., MAPK, PI3K, PLC, PKC, ion channels), depending on cell and tissue types. Tumors exploit the anti-inflammatory actions of extracellular adenosine to evade antitumor immune cells. A3R activation on mast cells promotes tumor microenvironment (TME) remodeling and angiogenesis, increases the population of M2 macrophages, and promotes the accumulation of myeloid-derived suppressor cells (MDSCs) in tumors. A2AR activation on T regulatory cells (Tregs) enhances their immunosuppressive activity (e.g., suppressing effector T cells). A2AR and/or A2BR activation on natural killer (NK) cells, dendritic cells, and effector T cells dampens the antitumor activity of these cells. Abbreviations: ectonucleoside triphosphate diphosphohydrolase-1 (CD39), ecto-5′nucleotidase (CD73), adenosine deaminase (ADA), phospholipase C (PLC), protein kinase C (PKC), diacylglycerol (DAG), phosphatidylinositol 4,5-bisphosphate (PIP_2_), inositol trisphosphate (IP_3_), mitogen-activated protein kinase (MAPK), phosphoinositide 3-kinase (PI3K).

Ecto-5′nucleotidase (*NT5E*; CD73) serves as a pacemaker for generating extracellular adenosine. With tissue damage, inflammation, and hypoxic stress, ATP is released from stressed, necrotic, and/or apoptotic cells and is hydrolyzed stepwise by ectonucleoside triphosphate diphosphohydrolase-1 (CD39), converting ATP to AMP, and CD73, converting AMP to extracellular adenosine (Figure 1). ATP's activation of ATP receptors promotes inflammation, whereas subsequent breakdown of ATP to extracellular adenosine and activation of adenosine receptors dampens inflammation (Figure 1) (45–47). Extracellular adenosine signals though four adenosine receptors: A1R, A2AR, A2BR, and A3R (48). The earliest link of extracellular adenosine to immunosuppression include studies on the anti-inflammatory activity of methotrexate (49) and seminal studies revealing A2AR signaling as essential in suppressing tissue-devastating inflammation (50). Extracellular adenosine protects tissues by dampening inflammation with myocardial injury (51–53), acute lung injury (54–58), intestinal ischemia-reperfusion injury (59–61), and inflammatory bowel disease (62–65). Tumors exploit extracellular adenosine' to protect the cancer cells. Extracellular adenosine accumulates in tumors and suppresses cytotoxic T cells and natural killer cells (66–68). Multiple studies using syngeneic and/or spontaneous tumor models show tumor growth and metastasis is significantly reduced by genetic deletion or pharmacological blockade of CD73 or A2AR; this effect is largely due to restoring antitumor immunity (30–44, 67–70). These mice also benefit from increased chemotherapy sensitivity (36, 71) and reduced angiogenesis (71, 72). In line with these studies, many human tumors overexpress CD73 and associates with poor prognosis (36, 73–78). CD73 is also linked to drug resistance, epithelial-to-mesenchymal transition (EMT), and cancer cell proliferation and stemness (76, 79–84). Tumors also grow slower in A2BR-deficient mice and mice treated with A2BR antagonists (85–87). For the most part, activation of A2AR and to a lesser extent A2BR on several types of immune cells, summarized below, promotes immunosuppression (Figure 1).

### Effector T Cells and T Regulatory Cells

A2AR is upregulated during inflammation on effector T cells. Its activation inhibits effector T cell proliferation, cytotoxic activity, and cytokine production [e.g., tumor necrosis factor-α (TNF-α), interferon-gamma (IFN-γ), interleukin-2 (IL-2)] (88–90). Whereas, A2AR activation on T regulatory (Treg) cells promotes Treg expansion and immunosuppressive activity [e.g., increasing forkhead box P3 (FoxP3) expression] (91). Mechanistically, these actions are linked together in a self-reinforcing loop. CD73 on Tregs generates extracellular adenosine and activates A2AR on effector T cells, suppressing effector T cell activity. Extracellular adenosine additionally activates A2AR on Tregs, promoting their expansion and activity (92). Human Tregs rarely express cell surface CD73 (93, 94), unlike mouse Tregs (92, 95). Instead, CD73 expression by surrounding cells or exosomes is considered to produce the extracellular adenosine. CD73 is expressed by populations of immune cells, stromal cells, epithelial and endothelial cells, cancer cells, and exosomes (96–98). Recently, CD39 co-expression with CD103 (integrin αE) was identified as a marker of antigen specific, tumor-reactive CD8+ T cells, having resident memory and a high capacity of recognizing and killing autologous tumor cells (99). These cells may be a strategy to improve adoptive cell therapy, which is limited by the ability to identify and expand tumor-reactive CD8+ T cells. Here, using CD39+ CD103+ to enrich the cells prior to *in vitro* expansion may increase therapy success (99). Many ongoing studies are directed at capturing and/or reinvigorating T cell-mediated antitumor responses. These studies will provide greatly to new approaches for extending and improving immunotherapy efficacy in cancer. A2AR activity also promotes peripheral T cell tolerance, skewing T cell differentiation from adaptive effector cells to adaptive FoxP3+ lymphocyte activation gene-3 (LAG-3)+ Tregs (100).

### Natural Killer Cells

A2AR activation on natural killer (NK) cells inhibits NK cell maturation, proliferation, activation, production of cytotoxic cytokines (e.g., IFN-γ and TNF-α), and target cell killing (38, 101–107). Whereas, genetic deletion or pharmacological blockade of A2AR or respiratory hyperoxia restores NK cell maturation, proliferative capacity, and cytotoxic function, which improves control over tumor growth, delays tumor initiation and suppresses tumor metastasis (38, 101, 102). CD73 and/or A2AR blockade or supplemental oxygen in combination with therapies promoting NK cell activity may be relevant strategies to enhance antitumor immunity. Whole-body exposure to 60% oxygen reduces tumor growth by reversing hypoxia-extracellular adenosine-mediated immunosuppression. In these preclinical studies, extracellular adenosine levels and CD39, CD73, A2AR, and A2BR gene expression decreases and coincides with increased antitumor immunity (102, 108). Hypoxia-inducible factors (HIFs) are strongly linked to increasing CD73 (109), A2AR (110), and A2BR (111) gene expression and collaborates to increase extracellular adenosine/adenosine receptor signaling for dampening inflammation (46, 47). Interestingly, recent studies show tumor cells can reprogram NK cells to gain immunosuppressive functions [e.g., increase IL-10 and transforming growth factor-β (TGF-β) production via signal transducer and activator of transcription 3 (STAT3) transcriptional activity, suppressing IFN-γ production] (112). The effects are not mediated through adenosine receptors, suggesting other mechanisms are involved and may not involve the production of extracellular adenosine (112).

### Myeloid-Derived Suppressor Cells and Tumor-Associated Macrophages

CD39 and CD73 are upregulated on CD11b+ CD33+ peripheral blood and tumor-associated myeloid-derived suppressor cells (MDSCs) via TGF-β, which their ectonucleotidase activity inhibits T cell and NK cell activity (113). Granulocytic MDSCs expressing high CD39 and CD73 are described in colorectal cancer patients. These cells were found to exert robust immunosuppressive features (e.g., high PD-L1 expression) and activity that could be dampened by blocking CD39/CD73 (114). A2BR activation preferentially promotes the expansion and intratumoral accumulation of CD11b+ Gr1+ MDSCs (115). CD11b+ Gr1+ MDSCs express high CD73, which limits T cell proliferation. CD73 is also considered to facilitate MDSC expansion by generating extracellular adenosine to activate A2BR on myeloid progenitors (115). Accordingly, blocking A2BR reduces CD11b+ Gr1+ MDSCs immunosuppression and accumulation in tumors (87). Extracellular adenosine generated by cancer cells can recruit tumor-associated macrophages (TAMs), which their endonucleotiase activity, in collaboration with CD73 expression on other cells of the tumor microenvironment, further contributes (e.g., suppressing antitumor CD4+ T cell proliferation) to extracellular adenosine-mediated immunosuppression in tumors (116).

### Dendritic Cells

Dendritic cells (DCs) transport tumor antigens to cytotoxic T lymphocytes for mounting antitumor immunity. A2BR activation on DCs inhibits their mobility, due to chemokine receptor downregulation, and they become tolerigenic to the tumor microenvironment (117–119). For instance, A2BR activation on DCs results in impaired allostimulatory activity and the expression of high levels of angiogenic, immunosuppressive, and tolerigenic factors [e.g., vascular endothelial growth factor (VEGF), IL-8, IL-6, IL-10, TGF-β, and idoleamine 2,3-dioxygenase (IDO)] (118). These cells cannot prime CD8+ T cells and T helper type 1 (Th1) immune responses (118–121). A2BR binding also inhibits monocyte differentiation to DCs (118, 119). A2BR blockade promotes DC activation (e.g., increased CD86 expression on CD11b- DCs), increases CD4+ and CD8+ T cell IFN-γ production, and tumor cell IFN-γ and CXCL10 expression (86), which supports the therapeutic potential of A2BR antagonists in enhancing antitumor immunity. Pharmacological agents for blocking A2BR are in clinical trials (e.g., NCT03274479; see Clinical implications section).

## Preclinical Studies Targeting CD73 and Adenosine Receptors

CD73's potential as an immunotherapy target has advanced rapidly within the last decade (30–44, 67–70). Current studies focus on combination strategies, including ICIs, adoptive transfer, chemotherapy, and targeted therapy. Preclinical studies show compelling evidence for both CD73 and A2AR blockade in enhancing anti-PD-1 and anti-CTLA-4 therapy. As single agents, both CD73 and A2AR blockade are effective in controlling tumor growth and metastasis. However, the combination of these therapies is far greater at reducing tumor growth, metastatic burden, and prolonging the life of mice. These effects depend on increased IFN-γ production and CD8+ T and NK cell activity (37, 38, 40, 43, 122, 123). Notably, anti-PD-1 therapy is particularly synergized by inhibiting CD73, (37) and studies report A2AR combined with anti-PD-1 therapy is most effective with cancer cells expressing high CD73. The latter suggests CD73 expression may stratify patients likely to benefit from anti-PD-1 therapy-A2AR blockade combination (122, 123). In melanoma, CD73 is a poor pretreatment biomarker for immunotherapy, however, its expression level in relapse tumors has predictive value (41). Therefore, CD73 as a biomarker may be tumor and sample (e.g., primary, metastasis, relapse) specific. Ciforadenant (formerly, CPI-444), an oral A2AR antagonist, recently completed a first-in-human study in patients with renal cell cancer (124). Preclinical studies have shown ciforadenant combined with anti-PD-L1 or anti-CTLA-4 therapy eliminates tumors in up to 90% of mice, restores antitumor immunity, and is effective in mice that failed prior anti-PD-L1 or anti-CTLA-4 therapy (68). Moreover, ciforadenant produced an antitumor memory response in which tumor growth was completely inhibited in mice with cleared tumors when later rechallenged (68). In clinical trials, cirforadenant combined with atezolizumab (anti-PD-L1 therapy) provide great disease control and survival benefit in patients, yet without high objective response rates (124). While reasons are unclear, the Fong and colleagues predict the response is due to persistent antitumor immunity that maintains durable control over tumor growth (124). Monotherapy ciforadenant also provided disease control in some individuals (124). Mechanistically, ciforadenant suppresses the expression of multiple checkpoint pathways on CD8+ effector T cells and CD4+ FoxP3+ Tregs and appears to have profound effects in restoring antitumor immunity at the draining lymph nodes by decreasing PD-1 and LAG-3 expression (69). Thus, a significant benefit of A2AR antagonism is its expansion of responsive cytotoxic T lymphocytes (69). A2AR and/or CD73 blockade also improves anti-CTLA-4 therapy efficacy in melanoma (43). Recently, anti-CD73 therapy combined with an agonist antibody to 4-1BB (4-1BB therapy) showed to restore antitumor immunity (125). 4-1BB is an activation-induced T cell costimulatory molecule that enhances cytotoxic T cell and NK cell activity (126, 127). 4-1BB therapy has entered into clinical trials involving GI cancer patients (NCT03330561). Poor efficacy and toxicity have been a concern in the past with 4-1BB therapy (128). Further preclinical studies are warranted.

Cancer vaccines educate the immune system to recognize cancer cells. Targeting A2AR in this setting also represents a promising strategy. Responses to melanoma and lymphoma tumor vaccines are increased in A2AR-deficient mice; these mice showed increased expansion of tumor-specific CD8+ T cells and increased survival compared to wild-type mice (129). The effectiveness of adoptive T cell transfer is also increased with genetic deletion or blockade of CD73 or A2AR. Tumor-bearing mice benefit from improved tumor control and survival due to increased infiltration and activation of adoptive T cells (30, 69, 70).

Additionally, preclinical studies show chimeric antigen receptor T (CAR T) cell efficacy is greatly increased by A2AR antagonism (130). CAR activation increases A2AR expression and suppression of mouse and human CAR T cells, which can be reversed by A2AR antagonism or genetic targeting, increasing the therapy benefit of CAR T cells. Efficacy is increased further by combination therapy (A2AR blockade and anti-PD-1 therapy) (130). Increased CD73 expression is seen in patients progressing under adoptive T cell transfer therapy (41). Accordingly, future approaches targeting CD73 in combination with A2AR blockade, anti-PD-1 therapy, and/or adoptive T cell transfer may prove beneficial. Head-to-head comparison studies blocking CD39 and CD73 (44) or CD73 and A2AR (39) also show promise for significantly increasing antitumor immunity. Co-targeting CD73 with A2AR inhibits the compensatory response of A2AR blockade to increase CD73 (39). Whereas, co-targeting CD39 with CD73 is beneficial by targeting two different mechanisms (44). Blocking CD39 elevates ATP levels. High ATP levels promote DC and macrophage antitumor activity, which adds to the antitumor immunity benefits of blocking CD73 (44). Combining CD73 anti-antibodies or small molecule inhibitors with chemotherapy or targeted therapies [e.g., antibodies against epidermal growth factor receptor (EGFR)] also shows merit in preclinical studies (36, 81). BRAF and MEK inhibitors combined with A2AR blockade show significant benefit in controlling melanoma tumor growth and metastasis in mice (42). A benefit of BRAF and MEK inhibitor combination is that it downregulates CD73 expression (42). Accordingly, this combination strategy provides the advantage of dampening CD73 expression without added drug/antibody therapy. Preclinical studies that focus on GI cancers will be essential in understanding the therapeutic potential of CD73 and/or adenosine receptor blockade in these tumors.

## Gastric Cancer

Gastric cancer (GC) is the fifth and third most common cancer and cause of cancer deaths worldwide, respectively (1). Although incidence and death rates are declining (131), advancements in prevention and treatment remain a priority. Five year survival rates drop to 20–30% or less once the cancer moves beyond the lining of the stomach (132). The majority of GC cases are advanced stage (133). Treatment includes gastric resection, radiation, chemotherapy, and targeted therapy, including antibodies against (VEGF)/VEGF receptor 2 (VEGFR2), and HER2 (131). Recently, ICI therapy was approved for GC (Table 1) (6). However, most patients do not benefit. Other immunotherapies being studied in GC include combination ICI therapy, adoptive cell transfer, vaccines [e.g., melanoma-associated antigen (MAGE) A3 peptides; Bacillus Calmette-Guerin (BCG)], and agonist antibodies for costimulatory receptors [e.g., OX40 (also known as tumor necrosis factor receptor superfamily, member 4), 4-1BB] (134).

Few studies have assessed CD73 expression in GC (Table 2). CD73 expression is higher in GC vs. normal tissue and associates with poor tumor differentiation, increased depth of invasion, positive nodal status, presence of metastasis, advanced-stage disease, and poor overall survival (Table 2) (75, 135). Increased CD73 in GC may be due in part to hypoxia. Hypoxia-inducible factor-1α (HIF-1α) staining closely correlates with high CD73 expression in gastric tumors (75). In contrast, gene expression studies have shown high CD73 expression associates with favorable overall survival in GC (136). Notably, CD73 expression does not always correlate to protein expression, which may explain the differences between these studies (136). Additionally, significant heterogeneity for CD73 is seen in GC (75, 135). For example, 30–50% of advanced stage, deeply invasive, and lymph node-positive tumors express low or no CD73 (75). Significant heterogeneity for CD73 expression is also described for melanoma (41, 42). In melanoma, CD73 expression is influenced by sample type (e.g., primary, metastatic, or relapse tissue); therapy treatment; and presence of activating MAPK (e.g., *BRAF*) mutations, mitogenic and inflammatory signals [e.g., hepatocyte growth factor (HGF) and TNF-α], and necrosis (41, 42). Increased *CD73* expression with *BRAF* mutation is also seen in serous ovarian cancer; these patients have better clinical outcomes (137). *BRAF* mutations are found in 10–20% of colorectal cancer and frequently are MSI-H (138–140). CD73 expression is also impacted by *NT5E* promoter methylation, described for both melanoma and breast cancer (141, 142). Suffice to say, multiple molecular and genetic factors can affect CD73 expression in human tumors.

**Table 2:** Summary of studies assessing CD73 expression in human GI cancers.

**Tumor type**	**Study**	**Findings**	**# of patients**	**Test method(s)**	**CD73 high advance stage tumors**	**Clinical significance**	**Reference (PMID)**
Gastric Cancer	Lu et al.	CD73 expression is higher in gastric cancer vs. normal tissue;High CD73 expression is positively correlated with tumor differentiation, histology, depth of invasion, nodal status, metastasis, American Join Committee on Cancer (AJCC) stage, and poor survival	68	IHC	50%	Poor prognosis	23569336
	Jiang et al.	High CD73 expression associates with favorable overall survival in gastric cancer;Meta-analysis study reports large heterogeneity for high CD73 expression for tumors (tumors: ovarian, breast, colorectal, gastric, gallbladder, prostate, rectal, renal, bladder, head and neck cancer, and NSCLC)	Oncomine database	mRNA, IHC (meta-analysis)	–	Better overall survival	29514610
	Hu et al.	CD73 expression is higher in gastric cancer vs. normal; High CD73 associates with advanced clinical stage, deep tumor invasion, lymph node metastasis, distant metastasis, and poor survival	408 (gastric cancer; TCGA)131 (gastric cancer; FFPE)	mRNA (TCGA), IHC, Western Blot	69%	Poor prognosis	30992388
Liver Cancer	Shrestha et al.	CD73 associates with poor overall survival and recurrence-free survival;Patients with tumors expressing high PD-L1 and high CD73 have poor prognosis	1,170 (combined datasets; GSE10143; GSE10186; GSE17856; TGCA Liver Cancer)	mRNA	–	Poor prognosis;Poor recurrence free survival in patients with high PD-L1	30057891
	Shali et al.	CD73 expression is higher in tumor vs. normal tissue;CD73 expression is positively correlated with epidermal growth factor receptor (EGFR) expression	30	IHC	–	–	30417547
	Ma et al.	CD73 expression is higher in hepatocellular carcinoma (HCC) vs. normal tissue;High CD73 expression correlates with microvascular invasion, poor differentiation increased time to recurrence, shorter overall survival, increased circulating tumor cells, and to epithelial-to-mesenchymal transition in HCC	232 (mixed: primary tumors, recurrence lesions, and metastases)	mRNA, IHC, Western Blot	57%	Poor prognosis	30971294
	Sciarra et al.	Immunohistochemistry study of CD73 expression in normal and hepatobiliopancreatic tissues;CD73 expression is present in all HCC, staining for CD73 ranges from intensity of 1+ to 3+ with a median intensity of 2+;Aberrant membranous and/or high/strong cytoplasmic expression for CD73 is seen in invasive HCC	24	IHC	CD73+ Staining Intensity = 3: 63%	–	30607549
	Snider et al.	*NT5E* is regulated by alternative splicing, producing a second transcript, *NT5E-2* in liver cirrhosis and HCC;*NT5E-2* is specific to humans and produces a protein product known as CD73 short (CD73s) that lacks enzyme activity (lacks exon 7) and is localized to the cytoplasm;*NT5E-2* is expressed at baseline in many normal human tissues;CD73s expression is 6–8-fold higher in HCC compared to normal liver tissues, whereas CD73 *(NT5E)* mRNA is dramatically deceased (>90%) in HCC	6 (HCC) 4 (Cirrhosis) 2 (Normal Liver)	mRNA, Immunofluorescence, Western Blot, Enzyme Activity	mRNA Expression HCC: *NT5E-2 =* 6–8-fold increase;*NT5E =* 90% decrease	Human specific isoform for CD73, *NT5E-2* (CD73s) that lacks enzyme activity CD73s increases in HCC, whereas CD73 decreases in HCC. CD73s is restricted to the cytoplasm	25298403
	Alcedo et al.	CD73 exhibits aberrant N-linked glycosylation in HCC cells and is independent of HCC etiology, tumor stage, or fibrosis presence. Aberrant glycosylation of CD73 results in a 3-fold decrease in enzyme activity;CD73 does not correlate with tumor immune subtype in HCC	HCC samples from PanCancer Atlas Consortium (mRNA) and 33 HCC (all other assays)	mRNA, Immunofluorescence, Western Blot, Enzyme Activity, Mass Spectrometry	CD73 Enzyme Activity: aberrant glycosylation of CD73 = 3-fold decrease in enzyme activity	CD73 is aberrantly glycosylated which significantly decreases its enzyme activity	31592495
Pancreatic Cancer	Zhou et al.	CD73 expression is higher in pancreatic ductal adenocarcinoma (PDAC) vs. normal tissues;High CD73 expression associates with increased tumor size, tumor stage, TMN stage, and poor prognosis	114	mRNA, IHC	40% (TMN stage)	Poor prognosis	30927045
	Sciarra et al.	Immunohistochemistry study of CD73 expression in normal and hepatobiliopancreatic tissues;CD73 is negative in acinar and islet epithelial cells, variable in pancreatic ducts, and mildly localized to stromal cells of normal and inflamed tissues;CD73 is expressed in 100% of PDAC;CD73 is expressed in a subset of pancreatic neuroendocrine neoplasms (PanNET/PanNEC) and almost absent in acinar cell carcinoma;Different staining patterns for CD73 are observed in PDAC, well- and moderately-differentiated tumors (grade 1 and grade 2) express apical CD73 staining similar to pancreatic ducts or express mixed membrane and cytoplasm staining; Poorly-differentiated PDACs express aberrant CD73 staining; PDAC, pancreatic ductal adenocarcinoma; MCA, mucinous cystadenoma; IPMN, intraductal papillary mucinous neoplasm; PanNET/PanNEC, pancreatic neuroendocrine tumor/pancreatic neuroendocrine carcinoma; ACC, acinar cell carcinoma	42 (PDAC) 5 (MCA) 13 (IPMN) 23 (PanNET/PanNEC) 19 (ACC)	IHC	CD73+ Staining Intensity = 3: 62% (PDAC) 0% (MCA) 0% (IPMN) 4% (PanNET/PanNEC) 5% (ACC)	PDAC: poor tumor differentiation and poor overall survival	30607549
	Katsuta et al.	PanNET/PanNEC express mild to moderate CD73 and associates with invasion into adjacent organs	44	IHC	54%	Invasion into adjacent organ	26691441
Colorectal Cancer	Wu et al.	CD73 expression is higher in colorectal cancer (CRC) vs. normal tissue;High CD73 expression associates with poor tumor differentiation, advanced tumor stage, metastasis, and poor overall survival	223 (cohort 1) 135 (cohort 2)	IHC, Western Blot	–	Poor prognosis	22287455
	Zhang et al.	CD73 expression in rectal cancer only samples;CD73 expression is increased in both tumor and stromal cells;High CD73 expression in cancer cells associates with poor patient prognosis;High CD73 expression in stromal cells associates with favorable characteristics (early T and tumor-node-metastasis (TMN) stages) and overall survival;Patients with high CD73 expression in both the cancer cells and stromal cells have similar good outcomes. No CD73 expression in both cell compartments is also favorable	90	IHC	–	High CD73 expression cancer cells = poor prognosis;High CD73 expression stromal cells = favorable outcomes	25677906
	Eroglu et al.	CD73 enzyme activity is higher in CRC vs. normal tissue;CD73 enzyme activity is higher in well-differentiated tumors compared moderately/poorly differentiated;No differences in CD73 enzyme activity seen with tumor stage, extent of invasion, metastasis, or tumor morphology	38	Enzyme Histochemistry	CD73 Enzyme Activity: CD73 enzyme activity is high in well-differentiated CRC compared to moderately and poorly differentiated CRC	CD73 enzyme activity high in tumors, associates with well-differentiated tumors	11114712
	Camici et al.	CD73 enzyme activity: no difference in CRC vs. normal tissue	16	Enzyme Histochemistry	–	No association	2125239
	Jiang et al.	Several types of tumors (cervical, liver, colorectal, prostate invasive ductal breast, small cell lung cancer and lung squamous cell carcinoma) showed similar CD73 expression vs. matched normal tissue	Oncomine database	mRNA, IHC (meta-analysis)	–	No association	29514610
	Cushman et al.	High CD73 expression associates with longer progression free survival from cetuximab (anti-EGFR therapy) in patients with KRAS-wild-type and mutant tumors.Epidermal growth factor receptor (EGFR)	103	mRNA	–	Biomarker for cetuximab (anti-EGFR therapy)	25520391

Looking ahead, assessing CD73 expression to common molecular and/or genetic alterations of GC and The Cancer Genome Atlas (TCGA) may help to better understand CD73 in GC (23). Studies assessing the association of CD73 expression to immune checkpoints, such as PD-L1, may also be helpful. Forty percent of GC cases are PD-L1 positive (143), and preclinical studies suggest high CD73 expression in PD-1/PD-L1 expressing tumors may identify patients that would benefit from combination anti-PD-1/PD-L1 therapy and CD73 and/or A2AR blockade (122, 123). Few studies globally assess CD73 expression with other ecto-enzymes involved in ATP and adenosine synthesis and metabolism and its intracellular uptake (144), such as other E-NTPDases, ecto-nucleotide pyrophosphatases/phosphodiesterases (e.g., CD203a), nitcotinamide dinucleotide enzyme (e.g., CD38), prostatic acid phosphatase, alkaline phosphatase (45, 145, 146), adenosine deaminase, and equilibrative and concentrative nucleoside transporters (ENTs and CNTs, respectively). Reviewed recently by Boison and Yegutkin (144), this may present a major gap in developing effective adenosine-based therapies (144). Accordingly, a more global view of extracellular adenosine metabolism and signaling in GC may also prove significant.

Considering CD73/extracellular adenosine's role in immune cell escape, studies of CD73's association to *H. pylori*-mediated tumorigenesis may provide additional insight. *H. pylori* infection is responsible for up to 60% of GC cases and arises in the background of inflammation (147, 148). Immune cell evasion is important for *H. pylori* infection and supported by evidence of higher PD-L1 expression in *H. pylori* positive compared to negative gastric biopsies (149) and that *H. pylori*-induced PD-L1 expression on gastric epithelial cells converts naïve T cells to CD4+ FoxP3+ Tregs that inhibit T cell proliferation (150). CD73 expression by CD4+ CD25+ Tregs enhances *H. pylori* infection by increasing local extracellular adenosine, which suppresses IFN-γ production (151). Consistent with this, infected CD73-deficient mice experience worse gastritis and more severe inflammation (e.g., increased IL-2, TNF-α, and IFN-γ and impaired Treg function) (151). Taken together, these studies support that CD73/extracellular adenosine in collaboration with other immune checkpoints may downregulate immune cell responses necessary for recognizing and clearing transformed cells arising in chronically infected gastric tissues, thus supporting GC development. With *H. pylori* infection, CagA and VacA containing exosomes are released from gastric epithelial cells, stimulating pro-inflammatory responses and affecting the expression of tumor suppressor and oncogenic genes (152). Considering CD73 expression on exosomes promotes tumor immunosuppression (97, 98), it would be interesting to see if CD73 is also expressed on *H. pylori*-mediated exosomes and if its presence or increased presence is a biomarker for the onset of GC.

Additional studies show CD73 promotes tumor cell proliferation, migration, invasion, and stemness in GC cells (135, 153). Antitumor roles for extracellular adenosine are also reported, including AMP-kinase (AMPK)-mediated, caspase-independent apoptosis, via intracellular uptake of extracellular adenosine through ENTs, and caspase-dependent apoptosis, mediated by A1R and A3R (154, 155). ENTs passively transport nucleosides based on a concentration gradient (Figure 1) (156, 157). A1R and A3R signaling both inhibit adenylyl cyclase activity and can activate multiple downstream signaling pathways, including phospholipase C, producing inositol 1, 4, 5-triphosphate (IP_3_) and diacylglycerol (DAG), mitogen-activated protein kinase (MAPK), and phosphoinositide 3-kinase (PI3K) (Figure 1) (158–160). A3R agonist, CF102, is in clinical trials for antitumor benefit in liver cancer (NCT02128958). A3R is also reported to increase HIF-1α through a non-transcription-dependent, non-HIF-1α oxygen-dependent degradation mechanism in several cancer cell lines (161). Though the role of A3R-mediated upregulation of HIF-1α is unclear, these data suggest A3R may both suppress and promote tumor progression. A2AR expression is increased in human GC tissue and correlates with poor tumor differentiation, advanced stage, lymph node positivity, and worse patient outcomes (162). Studies show A2AR, via PI3K-AKT-mTOR signaling, promotes GC cell stemness, EMT, and tumor cell migration and invasion (162). Altogether, more work is necessary to understand the role of CD73/extracellular adenosine in GC. Targeting specific adenosine receptors (e.g., A2AR) may be promising, but represents an area in need of more research.

## Liver Cancer

Liver cancer is the fourth most common cause of cancer death and sixth in terms of incidence worldwide (2). Ninety percentage of liver cancers are hepatocellular carcinoma (HCC) (163). Chronic liver disease (e.g., cirrhosis and fibrosis) is a major risk factor and most commonly caused by hepatitis B or C infection or long-term alcohol abuse (2, 164, 165). The 5-year survival rate for HCC is 18% (2). Treatment includes tumor resection, liver transplant, and targeted therapy (e.g., multi-kinase inhibitor, sorafenib) (166). However, 70% of patients do not qualify for surgery, due to advance disease, and sorafenib therapy is limited in its benefit; patient survival is prolonged only by a few months (166). ICI therapy was recently approved as second-line therapy for HCC (Table 1) (7, 9). Other promising immunotherapies are in development and are aimed at boosting existing or *de novo* immune responses, including vaccines and oncolytic viruses, and combination ICI therapy (167). Anticipation awaits the results of NCT03298451, a phase 3 clinical trial assessing anti-PD-L1 and anti-CTLA-4 combination therapy vs. monotherapy as better first-line options than sorafenib [HIMALAYA trial, (NCT03298451)].

In recent years, HCC has been a platform for the discovery of novel biology for CD73 in human tumors (Table 2) (168, 169). Studies by Snider and colleagues (168) identified an alternative splicing variant of *NT5E, NT5E-2* expressed in liver cirrhosis and HCC. *NT5E-2* produces a protein product, CD73-short (CD73s), and is a human-specific isoform that lacks enzyme activity and is unable to dimerize due to the loss of exon 7 with splicing (168). CD73s expression is limited to the cytoplasm and complexes with CD73 to promote proteasome-dependent degradation of CD73 (168). In HCC human tissues, CD73s expression is 6–8-fold higher compared to normal liver, whereas CD73 expression is downregulated by more than 90% (168). Accordingly, these studies indicate CD73s may be the major source of “CD73” overexpression in HCC. In contrast, other studies (170, 171) report CD73 is overexpressed in HCC and associates with poor tumor differentiation, microvascular invasion, and poor overall and recurrence-free survival (Figure 1) (170, 171). *NT5E-2* expression was not assessed in these studies, which is a limitation. In line with CD73s expression, Sciarra et al. (172) especially noted significant cytoplasmic CD73 expression in tumors, particularly with invasive tumors (172) (Table 2).

Many immunohistochemistry data for high CD73 expressing tumors, including gastric and pancreatic cancer, show significant cytoplasmic staining of CD73 (75, 77). Current commercial antibodies are not marketed to distinguish between CD73 and CD73s. Thus, other human tumors with CD73 overexpression may overexpress CD73s. Notably, *NT5E-2* is expressed at low levels in most normal human tissues and its expression increases with the onset of disease (123, 159). Currently, *NT5E-2* remains unstudied in other human tumors despite possible clinical implications. Similarly, recent studies by Alcedo et al. (169) report CD73 enzyme activity in HCC is significantly limited by aberrant glycosylation (169). The authors discovered that in HCC cells, unlike normal hepatocytes, CD73 carries abnormal N-linked glycosylation in its C-terminal catalytic domain, which greatly impairs the enzyme activity of CD73 (169). Aberrantly-glycosylated CD73 also showed to remain partially localized to the cytoplasm with golgi structural protein, GM130 (169). Importantly, these studies show that CD73 protein expression levels may not necessarily reflect its ability to generate extracellular adenosine. Studies by Snider et al. (168) and Alcedo et al. (169) are significant in that they demonstrate CD73 overexpression in human tumors can be misleading. Thus, CD73 immunohistochemistry may fall short in identifying patients likely to benefit the most from CD73 blockade therapy. As mentioned, commercial antibodies are unknown to be specific for recognizing CD73 vs. CD73s. Additionally, they are not primed for recognizing aberrant glycosylation. Instead, CD73 enzyme histochemistry is necessary, which is more challenging for clinical workups. These studies also raise questions as to how close preclinical studies of CD73/extracellular adenosine model human tumors. For instance, syngeneic and spontaneous mouse tumor models do not account for the biology of CD73s, which negatively regulates CD73 (168). A species-specific role of CD73 is also seen for arterial calcifications in humans and is not recapitulated in CD73-deficient mice (173, 174). CD73 downregulation in human tumors has been described in endometrial cancer. Its loss associates with more aggressive disease and poor overall survival (175). In normal endometrium, CD73-generated adenosine protects epithelial integrity, which CD73 loss and subsequently the loss of cell-cell adhesions promotes tumor progression (175). In contrast, in normal breast tissue, CD73 is expressed in myoepithelial cells as opposed to differentiated cells (e.g., acinar and ductal epithelial cells) (176). Myoepithelial cells are stem cell-like and exhibit highly invasive behavior similar to tumor cells (177). Consistent with this, CD73 is upregulated in cancer cells of triple-negative breast cancer (TNBC), which are tumors characterized by a gene expression signature similar to basal/myoepithelial cells (36, 177, 178). Accordingly, studies that reconcile tissue- and cell-specific roles for CD73 in normal GI tissues may help better understand CD73 in GI cancers (179, 180). Similar to endometrial cancer (175), CD73 is downregulated in cancer cells of bladder and prostate tumors and associates with poor prognosis (181, 182). The role of CD73 in bladder and prostate epithelium is unknown. Notably, CD39 deficiency promotes both induced and spontaneous autochthonous tumors in the liver (183).

For adenosine receptors, studies show A2AR signaling via PI3K-AKT promotes HCC tumor growth and metastasis and is reversed by A2AR antagonist treatment (170). A2BR expression is increased in human HCC tissue and correlates with tumor progression and is likely due to hypoxia (184). HIF-1α increases A2BR expression in HCC cells and cancer cell proliferation (184). Recent studies by Lan and colleagues (185) show HIF-1α's induced expression of A2BR is essential in enriching breast cancer stem cells for the onset of recurrent disease (185). Studies linking A2BR to tumor progression include work in bladder (86), breast (186), colon (187), and prostate (188) cancer and involves A2BR activity on both immune and tumor cells. A2BR antagonist, ATL801, reduces metastases by more than 80% in mice, which is due to increased IFN-γ, IFN-inducible chemokine CXCL10, a ligand for CXCR3, and tumor-infiltrating CXCR3+ T cells (86). Needless to say, interests in antagonizing A2BR in human tumors are rising. Studies by Vecchio et al. (188), in prostate cancer, describe a ligand-independent, constitutively active A2BR, which drives cancer cell proliferation (188). Importantly, these studies highlight an unappreciated view that adenosine receptors in tumors may not rely on CD73/extracellular adenosine. Aberrant ligand-independent G protein-coupled receptor constitutive activity is implicated in several cancers (188). In contrast, A3R expression is increased in human HCC and A3R promotes cancer cell apoptosis (189). A3R agonist, CF102, is being evaluated as second-line therapy for HCC (NCT02128958). Increased overall survival is reported with NCT02128958 and phase 3 studies are being planned (190). Taken together, adenosine receptors as opposed to CD73 may be better predictive targets for therapeutic benefit in HCC.

## Pancreatic Cancer

Pancreatic cancer is predicted to become the second leading cause of cancer-related deaths in the United States by 2030 (3, 4). Ninety percentage of pancreatic tumors are pancreatic ductal adenocarcinoma (PDAC) while 3–5% are neuroendocrine tumors (PNETs) (191). Smoking, heavy alcohol consumption, obesity, *H. pylori* infection, and chronic pancreatitis are risk factors (192). Prognosis is incredibly poor, approximately 70% of patients will succumb to the disease in the first year (193). The 5-year survival rate is 9% (193). Standard of care for pancreatic cancer includes radiation therapy, chemotherapy, and targeted therapy (e.g., EGFR inhibitors) (192). The prevalence of therapy resistance to these treatments is a persistent problem. PDAC patients have not benefited from single agent or combination ICI therapy (194–196) despite increased expression of PD-L1 in tumors (197–199). Significant efforts are underway to improve immunotherapy efficacy, including studies investigating regulatory B cell inhibition (e.g., Bruton's Tyrosine Kinase (BTK) inhibitors), IDO inhibition, and vaccine therapy (200). Though a predominant target in B cell malignancies, BTK in PDAC is shown to induce B cell- and macrophage-mediated T cell suppression, which BTK inhibitors (i.e., ibrutinib) restore T cell-dependent antitumor immunity and improve responsiveness to chemotherapy in preclinical studies (201). BTK inhibitors also produce an unexpected anti-fibrotic effect (202). PDAC cancers are rich in stromal cells and fibro-inflammatory reactions, which support chemotherapy resistance (203). A phase 3 clinical trial of ibrutinib in combination with chemotherapy in PDAC was recently completed (April 2019; NCT02436668) (204). Results are not yet publicly available.

Studies of CD73 in human PDAC tissue have only recently emerged (Table 2). CD73 is upregulated in PDAC compared to normal pancreatic tissue and correlates with increased tumor size, advanced stage, lymph node involvement, metastasis, and poor prognosis (77, 80, 172). While PDAC tumors are 100% positive for CD73 expression (172), interesting staining patterns for CD73 are seen. Well- and moderately-differentiated PDAC cells express mixed membrane and cytoplasmic CD73 staining. CD73 staining intensity is low to moderate in these tumors (172). In contrast, poorly-differentiated PDAC cells have aberrant CD73 staining, including very strong cytoplasmic CD73 expression (172). The increase of cytoplasmic CD73 expression in PDAC is unclear. We previously mentioned the discovery of CD73s in HCC (168). Studies assessing *NT5E-2* (CD73s) expression may help to better understand CD73 in PDAC. CD73 expression in acinar cell carcinomas (ACC) is rare (172). ACC comprises 1–2% of pancreatic tumors and does not carry typical genomic alterations seen in PDAC, including *KRAS* and *TP53* mutations (205), which is suggestive that CD73 expression in PDAC may be linked to *KRAS* and/or *TP53* mutations. *KRAS* mutation occurs in nearly 100% of PDAC cases (206). In human colorectal cancer (CRC) and non-small cell lung cancer (NSCLC) tissue, CD73 staining is increased in *KRAS* mutant compared to wild-type tumor (207). *KRAS* alterations associate with increased CD73, CD39, A2AR, and A2BR gene expression in CRC and NSCLC cell lines, which correlates with anti-PD-1 resistance in *KRAS* mutant tumor models (207). Moreover, high CD73 expression and *KRAS* alterations associate with worse overall survival compared to patients with *KRAS* alterations and low CD73 expression tumors (207). EGFR alterations and high CD73 expression also associate with poor overall survival (TCGA pan-cancer) (207). EGFR alterations and *KRAS* mutations occur together in 67% of PDAC cases (208). Accordingly, EGFR alterations may also increase CD73 expression in PDAC. A positive association between CD73 expression and EGFR alterations is described in breast cancer (209).

CD73 expression (3+ staining) increases with aggressive disease in PDAC (172), which may be an indicator of an evolving or advancing immunosuppression phenotype. For instance, in PDAC, a decrease in CD8+ T cell infiltration into tumors is seen with the rise of infiltrating Tregs with disease progression (210). As mentioned, human Tregs rarely express cell surface CD73 (93, 94), and it is considered that CD73-generated extracellular adenosine from other sources [e.g., cells (96) or exosomes (97, 98)] activate adenosine receptors on immune cells for immunosuppression. Accordingly, the coinciding increase of CD73 in PDAC cells may be significant in promoting extracellular adenosine-mediated immunosuppression. Other cells and cell-derived products possibly contributing are CD4+ CD73+ T cells, B cells, and CD39+ CD73+ exosomes (211). CD73+ PDAC and NSCLC cell-derived exosomes activate A3R on intratumor and peripheral mast cells, which promotes remodeling of the tumor microenvironment through increasing the expression of angiogenic factors (212, 213). Additionally, in PDAC models, tumor-infiltrating CD11b+ CD103– DCs promote tumor growth by inducing expansion of FoxP3^neg^ CD39+ CD73+ tumor-promoting Tregs (214).

Pancreatic neuroendocrine tumors and carcinomas (PanNET/PanNEC) account for 1–10% of pancreatic tumors (215, 216). Thirty to fifty percent of PanNET/PanNEC express mild to moderate CD73 expression and associates with increased malignant potential, which is similar to gastrointestinal (GI)-NET/NECs (80, 172, 217). In GI-NET/NECs, CD73 expression positively correlates with PD-L1 expression (217), which possibly anti-PD-1/PD-L1 therapy with CD73 and/or A2AR blockade may benefit these patients. Increased expression of CD73 with PanNET also associates with cancer cell stemness (e.g., aldehyde dehydroxygenase expression) and aggressive behavior (80). Filippini et al. (218) recently reported a transplantable model of mouse pancreatic tumor organoids into immunocompetent mice that recapitulate human PDAC progression and that the system serves as a suitable model for immunophenotypic studies (218). The organoid-derived isographs induce the expression of many immunosuppressive/aggressive biomarkers with tumor development and evolution, including CD73 (218). Studies using such models may provide a significant understanding of CD73/extracellular adenosine signaling in immunosuppression and the immunoevolution of PDAC.

CD73 also shows to promote drug resistance and tumor growth in PDAC cells. For instance, high CD73 expression and low miR-30a-5p expression in PDAC cells result in chemotherapy (e.g., gemcitabine) resistance (77), and CD73 knockdown inactivates AKT and extracellular signal-regulated kinase (ERK) signaling and slows cancer cell growth (77). In contrast, studies show extracellular adenosine treatment in combination with AKT inhibitor, GSK690693, reduces PDAC growth and induces tumor cell apoptosis and senescence in patient-derived xenografts (PDX). Mechanistically, the intracellular uptake of extracellular adenosine via ENTs (Figure 1) appears important for this response, as dipyridamole (pan-ENT inhibitor) treatment remarkably recovers cell viability (219). The difference between these studies likely relates to the subcutaneous transplanting of tumors (77) vs. tumors transplanted to the tail of the pancreas (219). Indeed, for example, CD39 deficiency can promote the development of both induced and *de novo* tumors in the liver, which is in contrast to its role in antitumor immunity of subcutaneous transplanted tumors (183, 220). It is considered that the surrounding microenvironment and interaction with these cells by the tumor likely produce different responses and outcomes. In the next several years, adopting in-depth and detailed characterization of CD73/extracellular adenosine in immunocompetent, autochthonous pancreatic cancer models, humanized models, and human organoids will be essential for better understanding the possible therapeutic benefit of targeting CD73 and adenosine receptors in pancreatic tumors.

## Colorectal Cancer

Colorectal cancer (CRC) is the third most common cancer and the second cause of cancer-related deaths worldwide (5). CRC incidence rates are declining in the United States and are stable in most other Western countries, whereas rates are rising in Eastern Asia and Eastern Europe and likely reflect the adoption of a Western lifestyle (221, 222). CRC risk factors include obesity, Western diet, lack of physical activity, excessive alcohol use, hereditary syndromes (e.g., Lynch syndrome), and smoking (223). Treatment includes surgery, combination chemotherapy, radiation therapy, and targeted therapy, including antibodies against VEGF/VEGFR or EGFR (224). Although advances in better screening and treatment have been made in the last decade, long-term survival remains poor for metastatic CRC patients. The 5-year survival rate is <15% (225). ICI therapy was recently approved for refractory dMMR/MSI-H metastatic CRC (Table 1) (8, 10, 11). Of CRC cases that are dMMR/MSI-H, only 4% are metastatic. Accordingly, several approaches, including IDO inhibitors, vaccine therapy, and combination ICI therapy are being studied to extend immunotherapy efficacy to more patients (226). A better understanding of CD73/adenosine receptor signaling in CRC may help in these efforts.

Early studies assessing CD73 in CRC were part of larger efforts examining enzymatic patterns of key enzymes involved with purine metabolism and salvage, including ADA, alkaline phosphatase, hypoxanthine-guanine phosphoribosyltransferase (Table 2) (227, 228). Studies by Camici et al. (227) reported no difference with CD73 enzyme activity between CRC and normal tissue (227). In contrast, Eroglu et al. (228) showed higher CD73 enzyme activity in tumors compared to normal tissue (228). No associations were found with high CD73 enzyme activity and poor clinical features. Instead, high CD73 enzyme activity was associated with well-differentiated tumors and low CD73 enzyme activity associated with poorly-differentiated tumors (228). More recent studies show high CD73 expression correlates with poor tumor differentiation, lymph node involvement, advanced stage, and poor survival (78). In rectal cancer, CD73 expression in the different cell types carries different clinical prognosis (229). High CD73 expression in cancer cells and low CD73 expression in stromal cells associates with poor overall survival, whereas low CD73 expression in cancer cells and high CD73 expression in stromal cells is more favorable (229). Bladder cancer is similar. CD73 positive expression by epithelial cells predicts better progression-free survival and overall survival, whereas stromal cell CD73 positivity predicts poor outcome (230). Accordingly, these studies support that CD73 in tumors may suppress and promote tumor progression. Although unknown, targeting tumors with dual roles for CD73 may prove challenging for CD73 inhibitor therapy.

Tumor heterogeneity is likely one explanation for the reported differences of CD73 expression in CRC. CRC tumors carry significant inter- and intra-heterogeneity (231, 232), so much so that in recent years an international consortium was formed to establish a robust molecular and genetic classification scheme for CRC. These global efforts led to the development of the consensus molecular subtypes (CMS) (233). Accordingly, future efforts assessing CD73 expression to the CMS groups (e.g., CMS1, CMS2, CMS3, CMS4) may provide a better understanding of CD73 in CRC and the possible molecular and genetic alterations that drive its downregulation and/or overexpression. Indeed, high CD73 expression in CRC may be associated with CMS2 tumors. In CRC, CD73 is a predictive biomarker of patient response to anti-EGFR therapy (234). In line with this, the CMS2 group predicts tumors that are more responsive to anti-EGFR and anti-HER2 therapy (235). Also consistent is that CD73 promotes CRC cell proliferation and tumor growth through β-catenin (WNT)/cyclin D1 signaling (236) and CMS2 tumors are characterized by WNT and MYC signaling (233). *KRAS* mutations/alterations are likely also linked to CD73 expression in CRC; discussed previously in the section on PDAC (207). Thus, investigating CD73 expression in *KRAS* mutant tumors may provide additional insight. A focus on metastatic samples may also be important. Liver metastasis occurs in 50% CRC patients (237). Recently, studies have shown high CD73 expression associates with significantly shorter time to recurrence and poor survival (238). In renal cancer patients, an adenosine high (AdenoSig^hi^) expression signature was identified in pretreatment biopsies and associated with clinical response to A2AR antagonism (124). Similar efforts in identifying biomarker signatures may provide greatly to improving immunotherapy efficacy in CRC.

In preclinical studies, CD73 deletion increases CD8+ T cells and IFN-γ production to suppress the growth of MC-38 mouse colon cancer (32). The depletion of CD73 on CD4+ Foxp3+ Tregs also is significant in restoring antitumor immunity in this model (32). Similarly, CD39-deficient mice are resistant to MC-38 metastasis (239, 240). Whereas, overexpression of CD39 increases MC-26 mouse colon cancer cell metastasis to the liver (220). CD39 deletion does not increase the development of primary MC-26 orthotopic transplant tumors in heterozygous CD39 mice or mice transgenic for human CD39 compared to wild-type mice (220). Recent studies show support for co-targeting CD39 and CD73 in combination with ICI therapy and/or chemotherapy (44). Tumor-bearing mice benefit from increased antitumor immunity in these studies, which is due to the recovery of DC, macrophage, and effector T cell antitumor activity (44). In line with these studies, inhibiting CD39 or CD73 on MDSCs from CRC patients is effective in dampening the immunosuppressive activity of these cells (114).

Adenosine receptors may also be possible therapeutic targets. High A2AR expression associates with larger tumor size, increased tumor invasion, and higher TNM (TNM Classification of Malignant Tumors) stage in CRC (241). High A2AR expression also predicts poor patient survival and is positively correlated with PD-L1 expression (241). Consistent with the possible benefit of combined A2AR antagonist and ICI therapy (43, 68, 122, 123), studies with MC-38 cells, show A2AR antagonist, ciforadenant, combined with anti-PD-L1 or anti-CTLA-4 therapy eliminates 90% of tumors in mice by restoring antitumor immunity (68). Notably, MC-38 cells are normally highly sensitive to ICI therapy (37, 123). Additionally, in many studies, MC-38 cells are grown subcutaneously. Accordingly, it is not known how close these preclinical studies model immunosuppression and immunotherapy efficacy for CRC. A2BR is also upregulated in CRC and likely is linked to tumor hypoxia and progression (187). *In vitro* studies show A2BR expression is upregulated in CRC cells by hypoxia and promotes cancer cell proliferation, which is dampened by A2BR antagonism (187). A2BR antagonism also dampens A2BR-mediated CD73 expression by cancer-associated fibroblasts (CAFs) and CAF-associated immunosuppression activity (242). A3R is overexpressed in human CRC tissue and stimulates tumor growth via extracellular signal-regulated protein kinases 1 and 2 (ERK1/2) (243, 244). In contrast, studies also report A3R activity inhibits tumor growth by modulating glycogen synthesis kinase-3β (GSK-3β) and NF-Kappaβ (NF-κβ). A3R agonist treatment inhibits CRC cell proliferation, limits liver metastasis, and increases the cytotoxicity of chemotherapy (e.g., 5-fluorouracil) (245–247). Interestingly, treatment of CRC cells with caffeine, a non-selective adenosine receptor antagonist, inhibits A3R-mediated stabilization of HIF-1α (248). It is unclear if HIF-1α mediated by A3R promotes tumor progression or antitumor activity. HIFs are described to have pro- and antitumor activity in CRC (249). Moreover, overexpression of HIF-1α does not increase CRC tumorigenesis and does not result in spontaneous tumor formation in mice (250). Taken together, while many adenosine pathway members show evidence for possible therapeutic targeting in CRC, detailed studies in human tumors and relevant preclinical models are greatly needed.

## Clinical Implications

Inhibiting CD73 (and/or A2AR) restores antitumor immunity in many preclinical studies with combination approaches showing superior efficacy. Accordingly, several clinical trials inhibiting CD73 (e.g., antibodies against CD73 or small molecule inhibitors) in combination with ICI therapy, A2AR antagonism, targeted therapy, and/or chemotherapy are underway (Table 3). Preliminary safety profiles report BMS-986179, an anti-CD73 humanized monocolonal antibody, and its combination with nivolumab (anti-PD-1 therapy) to be well-tolerated in patients (NCT02754141) (251). Recent studies in renal cell cancer (RCC) reported the feasibility and safety of A2AR antagonist, ciforadenant (124). Similar to preclinical studies, durable clinical benefit was associated with increased recruitment of CD8+ T cells (124). Additionally, combination therapy (ciforadenant and anti-PD-L1 therapy) showed benefit in patients who had progressed on anti-PD-1/PD-L1 therapy. Notably, patients in these trials were heavily pretreated (≥3 prior treatments) (124). It will be interesting in the future to see if CD73 and/or A2AR therapy efficacy is increased further when used in earlier lines of therapy (124). Moreover, the authors discovered responding patients carry an AdenoSig^hi^ signature (124). Assessing whether this signature can also be detected in pretreatment biopsies of other cancers and possibly primary tumors may be beneficial (124). Biomarkers or gene signatures will likely be key in identifying patients benefiting the most from CD73/adenosine receptor therapy. Clinical trials are underway for AB928, a dual A2AR/A2BR antagonist, and include a focus on GI cancers [e.g., esophageal cancer and CRC; NCT03720678 (Table 3)]. A favorable safety profile of AB928 combined with chemotherapy has been reported in patients (252). Future studies in GI cancers that focus on determining if adenosine-mediated resistance to immunotherapy therapy exists at diagnosis or evolves with therapy will also be of significant benefit. Encouraging early results for BMS-986179 combined with nivolumab report clinical benefit (partial response) in one or more patients with pancreatic and prostate cancer (NCT02754141) (251). Both are poorly immunogenic tumors. Preclinical studies show CD73/adenosine therapy (e.g., A2AR deletion) liberates CD8+ T cells for antitumor activity even against weakly immunogenic sarcomas (70). Therapy benefit in these studies is independent of the anatomical location of the tumor (70). Thus, therapeutic benefit across many tumors (immunogenic and non-immunogenic) is expected. Understanding factors preventing immune cells from recognizing and eliminating cancer cells will continue to be important in the advancement of immunotherapy strategies. Poor tumor immunogenicity can be a result of many features, including HLA class I molecule downregulation or loss (253); genetic, epigenetic, and chromosome alterations regulating presentation and processing of surface epitopes (254–256); expression and secretion of immunosuppressive factors (e.g., PD-1, TGF-β, adenosine) (257); and the inability of cancer cells to produce new surface epitopes that are different from what immune receptors have regularly experienced (258). Whether CD73 expression associates with dMMR/MSI-H in GI tumors and its blockade would further increase immunotherapy efficacy in these tumors is unknown. In NSCLC studies, tumor mutational burden and neoantigen burden does not associate with CD73 high or low expression (74).

**Table 3:** Summary of clinical trials for CD73, A2AR, and A2BR in cancer.

**Adenosine pathway target**	**Drug(s)**	**Target(s)**	**Therapy modality (adenosine pathway)**	**Phase**	**Details**	**Disease**	**Status**	**ClinicalTrials.gov Identifier**
CD73	LY3475070 Pembrolizumab	CD73PD-1	LY3475070:CD73 SmallMolecule Inhibitor	Phase 1	Cohort A: LY3475070 administered orallyCohort B: LY3475070 + Pembrolizumab administered IVCohort C1: LY3475070 + Pembrolizumab administered IVCohort C2 LY3475070 administered orallyCohort D1 LY3475070 + Pembrolizumab administered IVCohort D2: LY3475070 administered orallyCohort E: LY3475070 + Pembrolizumab administered IV	Advanced SolidMalignancies	Recruiting	NCT04148937
	Oleclumab (MEDI9447) Durvalumab (MEDI4736)	CD73PD-L1	Oleclumab: CD73 Humanized Monoclonal Antibody	Phase 1 Phase 2	Phase I and Phase II Arm A: Paclitaxel, Carboplatin, Durvalumab, + OleclumabPhase II Arm B: Paclitaxel, Carboplatin, + Durvalumab	Triple NegativeBreast Cancer	Recruiting	NCT03616886
	Oleclumab (MEDI9447) Durvalumab	CD73PD-L1	Oleclumab:CD73 HumanizedMonoclonalAntibody	Phase 2	Experimental: Chemotherapy and radiation Experimental: Chemotherapy and pre-operative radiotherapy + Durvalumab Experimental: Chemotherapy and pre-operative radiotherapy + Durvalumab and Oleclumab	Luminal B(Breast Cancer)	Recruiting	NCT03875573
	Oleclumab(MEDI9447)Durvalumab	CD73PD-L1	Oleclumab:CD73 HumanizedMonoclonalAntibody	Phase 1	Experimental: Monotherapy, Oleclumab Experimental: Combination, Oleclumab and Durvalumab	Solid Tumors	Active, not Recruiting	NCT02503774
	TJ004309Atezolizumab	CD73PD-L1	TJ004309:CD73 HumanizedMonoclonalAntibody	Phase 1	Dose escalated TJ004309 + Atezolizumab	Solid TumorsMetastatic Cancer	Recruiting	NCT03835949
	Oleclumab (MEDI9447) Durvalumab AZD9150 AZD6738 Vistusertib Olaparib Trasutzumab Cediranib	CD73 PD-L1 STAT3 ATR mTOR PARP HER2 VEGFR	Oleclumab: CD73 Humanized Monoclonal Antibody	Phase 2	Experimental: Durvalumab + Olaparib Experimental: Durvalumab + AZD9150 Experimental: Durvalumab + AZD6738 Experimental: Durvalumab + Vistusertib Experimental: Durvalumab + Oleclumab Experimental: Durvalumab + Trastuzumab Experimental: Durvalumab + Cediranib	Non-Small Cell Lung Cancer	Recruiting	NCT03334617
	Oleclumab (MEDI9447) Durvalumab Capivasertib Danvatirsen Paclitaxel	CD73 PD-L1 AKT STAT3 Chemotherapy	Oleclumab: CD73 Humanized Monoclonal Antibody	Phase 1 Phase 2	Experimental: Durvalumab + Paclitaxel Experimental: Durvalumab + Paclitaxel + Capivasertib Experimental: Durvalumab + Paclitaxel + Danvatirsen Experimental: Durvalumab + Paclitaxel + Oleclumab	Triple Negative Breast Cancer	Recruiting	NCT03742102
	Oleclumab (MEDI9447) Durvalumab Gemcitabine Nab-paclitaxel Oxaliplatin Leucovorin 5-FU	CD73 PD-L1 Chemotherapy	Oleclumab: CD73 Humanized Monoclonal Antibody	Phase 1 Phase 2	Arm A1: Gemcitabine + Nab-paclitaxelArm A2: Oleclumab + Gemcitabine + Nab-paclitaxelArm A3: Oleclumab + Durvalumab + Gemcitabine/Nab-paclitaxelArm B1: Oxaliplatin + Leucovorin + 5-FU (mFOLFOX)Arm B2: Oleclumab + mFOLFOX	CarcinomaMetastatic PancreaticAdenocarcinoma	Active, notRecruiting	NCT03611556
	Oleclumab (MEDI9447) Durvalumab	CD73PD-L1	Oleclumab:CD73 HumanizedMonoclonal Antibody	Phase 1	Experimental: Monotherapy, Durvalumab Experimental: Combination, Durvalumab + Oleclumab	Muscle InvasiveBladder Cancer	Recruiting	NCT03773666
	BMS-986179 Nivolumab rHUPH20	CD73PD-1 Hyaluronidase	Oleclumab: CD73 Humanized Monoclonal Antibody	Phase 1 Phase 2	Arm A: Monotherapy, BMS-986179Arm B: Combination Therapy, BMS-986179 + NivolumabArm C: Combination Therapy, BMS-986179 + rHUPH20	Malignant Solid Tumor	Recruiting	NCT02754141
	Oleclumab (MEDI9447) MEDI0562 Durvalumab Tremelilumab	CD73 OX40 PD-L1 CTLA-4	Oleclumab: CD73 Humanized Monoclonal Antibody	Phase 2	Cohort A: Oleclumab + DurvalumabCohort B: MEDI0562 + DurvalumabCohort C: MEDI0562 + Tremelimumab	Ovarian Cancer	Recruiting	NCT03267589
CD73 A2AR	CPI-006 Ciforadenant (CPI-444) Pembrolizumab	CD73 A2AR PD-1	CPI-006: CD73 Humanized Monoclonal Antibody Ciforadenant: A2AR Antagonist	Phase 1	Cohort 1a: (escalating doses) CPI-006Cohort 1b: (escalating doses) CPI-006 + CiforadenantCohort 1c: (escalating doses) CPI-006 + PembrolizumabCohort 2a: (selective dose) CPI-006Cohort 2b: (selective dose) CPI-006 + CiforadenantCohort 2c: (selective doses) CPI-006 + Pembrolizumab	Non-Small Cell Lung Cancer Renal Cell Cancer Colorectal Cancer Triple Negative Breast Cancer Cervical Cancer Ovarian Cancer Pancreatic Cancer Endometrial Cancer Sarcoma Squamous Cell Carcinoma of the Head and Neck Bladder Cancer Metastatic Castration Resistant Prostate Cancer Non-hodgkin Lymphoma	Recruiting	NCT03454451
	Oleclumab (MEDI9447) AZD4635 Durvalumab	CD73 A2AR PD-L1	Oleclumab: CD73 Humanized Monoclonal Antibody AZD4635: A2AR Antagonist	Phase 2	Module 1: Drug: AZD4635; Drug: DurvalumabModule 2: Drug: AZD4635; Drug: Oleclumab	Prostate Cancer Metastatic Castration-Resistant Prostate Cancer	Recruiting	NCT04089553
	Oleclumab (MEDI9447) AZD4635 Osimertinib	CD73 A2AR EGFR	Oleclumab: CD73 Humanized Monoclonal Antibody AZD4635: A2AR Antagonist	Phase 1 Phase 2	Arm A: MEDI9447 + OsimertinibArm B: MEDI9447 + AZD4635	Non-Small Cell Lung Cancer	Recruiting	NCT03381274
	NZV930NIR178PDR001	CD73 A2AR PD-1	NZV930: CD73 Humanized Monoclonal Antibody NIR178: A2AR Antagonist	Phase 1	Experimental: NZV930 Experimental: NZV930 + PDR001 Experimental: NZV930 + NIR178 Experimental: NZV930, NIR178, PDR001	Non-small Cell Lung Cancer (NSCLC) Triple Negative Breast Cancer Pancreatic Ductal Adenocarcinoma Colorectal Cancer Microsatellite Stable Ovarian Cancer Renal Cell Carcinoma	Recruiting	NCT03549000
	Oleclumab (MEDI9447) AZD4635 Durvalumab Abiraterone Acetate Enzalutamide Docetaxel	CD73 A2AR PD-L1 Hormone Therapy Chemotherapy	Oleclumab: CD73 Humanized Monoclonal Antibody AZD4635: A2AR Antagonist	Phase 1	Experimental: Arm A: AZD4635 monotherapy as nanoparticle suspension 125 mg BID Experimental: Arm B: AZD4635 monotherapy as nanoparticle suspension 75 mg QD Experimental: Arm C: AZD4635 monotherapy as nanoparticle suspension 100 mg QD Experimental: Arm D: AZD4635 as nanoparticle suspension 75 mg QD plus Durvalumab Experimental: Arm E: AZD4635 as nanoparticle suspension 100 mg QD plus Durvalumab Experimental: Arm EA: AZD4635 as nanoparticle suspension plus Enzalutamide Experimental: Arm AA: AZD4635 as nanoparticle suspension plus Abiraterone Acetate Experimental: Arm F: AZD4635 as nanoparticle suspension plus Durvaluamb in patients post immunotherapy with non-small cell lung cancer Experimental: Arm G: AZD4635 monotherapy as nanoparticle suspension in patients post immunotherapy with non-small cell lung cancer Experimental: Arm H: AZD4635 monotherapy as nanoparticle suspension in patients post immunotherapy with other solid tumors Experimental: Arm I: AZD4635 as nanoparticle suspension plus Durvalumab in immunotherapy naïve patients with metastatic castration resistant prostate cancer Experimental: Arm J: AZD4635 as nanoparticle suspension plus Durvalumab in immunotherapy naïve patients with metastatic castration resistant prostate cancer	Advanced Solid Malignancies Non-Small Cell Lung Cancer Metastatic Castrate-Resistant Prostate Carcinoma Colorectal Carcinoma	Recruiting	NCT02740985
					Experimental: Arm K: AZD4635 monotherapy as nanoparticle suspension in immunotherapy naïve patients with colorectal carcinoma Experimental: Arm KD: AZD4635 as nanoparticle suspension plus Durvalumab in immunotherapy-naïve patients with colorectal carcinoma Experimental: Arm L: AZD4635 monotherapy as nanoparticle suspension in immunotherapy naïve patients with other solid tumours Experimental: Arm CA: AZD4635 capsule formulation monotherapy 75 mg QD Experimental: Arm CB: AZD4635 capsule formulation 50 mg QD plus Durvalumab and Oleclumab Experimental: Arm CC: AZD4635 capsule formulation 50 mg QD plus Docetaxel			
A2AR	NIR178PDR001	A2ARPD-1	NIR178: A2AR Antagonist	Phase 2	Experimental (1): NIR178 + PDR001 Experimental (2): NIR178 BID Intermittent + PDR001 Experimental (3): Part 3, initiation of part 3 will depend on results from parts 1 and 2 Experimental (4): Japanese safety run-in part, two different dosing schedules of NIR178 will be explored	Non-small Cell Lung Cancer Renal Cell Cancer Pancreatic Cancer Urothelial Cancer Head and Neck Cancer Diffused Large B Cell Lymphoma Microsatellite Stable Colon Cancer Triple Negative Breast Cancer Melanoma	Recruiting	NCT03207867
	PBF-509PDR001	A2ARPD-1	PBF-509: A2AR Antagonist	Phase 1 Phase 2	Drug: PBF-509_80 mgDrug: PBF-509_160 mgDrug: PBF-509_320 mgDrug: PBF-509_640 mgDrug: Combo PBF-509 (160 mg) + PDR001Drug: Combo PBF-509 (320 mg) + PDR001Drug: Combo PBF-509 (640 mg) + PDR001Drug: RP2D (PBF-509+PDR001)_immuno naïveDrug: Experimental: RP2D (PBF-509+PDR001)_immuno treated	Non-small Cell Lung Cancer	Recruiting	NCT02403193
	NIR178 Spartalizumab LAG525 Capmatinib MCS110 Canakinumab	A2ARPD-1LAG-3c-MetM-CSFIL-1β	NIR178: A2AR Antagonist	Phase 1	Experimental: Spartalizumab + LAG525 + NIR178 Experimental: Spartalizumab + LAG525 + Capmatinib Experimental: spartalizumab + LAG525 + MCS110 Experimental: spartalizumab + LAG525 + Canakinumab	Triple Negative Breast Cancer	Recruiting	NCT03742349
	Ciforadenant (CPI-444) Atezolizumab	A2AR PD-L1	Ciforadenant: A2AR Antagonist	Phase 1	Experimental: Ciforadenant, 100 mg orally twice daily for the first 14 days of each 28-day cycle Experimental: Ciforadenant, 100 mg orally twice daily for 28 days of each 28-day cycle Experimental: Ciforadenant, 200 mg orally once daily for the first 14 days of each 28-day cycle Experimental: Ciforadenant + Atezolizumab Experimental: Ciforadenant, start with 150 mg orally twice daily for 28-day cycles; then, increase increments by 100 mg/day for 6 dose levels	Non-Small Cell Lung Cancer Malignant Melanoma Renal Cell Cancer Triple Negative Breast Cancer Colorectal Cancer Bladder Cancer Metastatic Castration Resistant Prostate Cancer	Recruiting	NCT02655822
	Ciforadenant (CPI-444) Atezolizumab Cobimetinib RO6958688 Docetaxel Pemetrexed Carboplatin Gemcitabine Linagliptin Tocilizumab Ipatasertib Idasanutlin	A2AR PD-L1 MEK CEA Chemotherapy IL-6R AKT MDM2	Ciforadenant: A2AR Antagonist	Phase 1 Phase 2	Active Comparator: Stage 1: Cohort 1: Atezolizumab Experimental: Stage 1: Cohort 1: Atezolizumab + Cobimetinib Experimental: Stage 1: Cohort 1: Atezolizumab + RO6958688 Active Comparator: Stage 1: Cohort 2: Docetaxel Experimental: Stage 1: Cohort 2: Atezolizumab + Cobimetinib Experimental: Stage 1: Cohort 2: Atezolizumab + Ciforadenant Experimental: Stage 1: Cohort 2: Atezolizumab + RO6958688 Experimental: Stage 1: Cohort 2: Atezolizumab + Ipatasertib Experimental: Stage 1: Cohort 2: Idasanutlin + Docetaxel Experimental: Stage 2: Cohort 1: Atezolizumab + Pemetrexed + Carboplatin	Carcinoma, Non-Small-Cell Lung	Recruiting	NCT03337698
A2AR A2BR	AB928 IPI-549 Doxorubicin Paclitaxel	A2AR/A2BR PI3Kγ Chemotherapy	AB928: Dual A2AR and A2BR Antagonist	Phase 1	Experimental: Dose Escalation-Arm A, AB928 + Pegylated Liposomal Doxorubicin Experimental: Dose Escalation-Arm B, AB928 + Nanoparticle Albumin-bound Paclitaxel Experimental: Dose Escalation-Arm C, AB928 + Pegylated Liposomal Doxorubicin + Nanoparticle Albumin-bound Paclitaxel Experimental: Dose Expansion-TNBC-Arm 1, dose from Arm A for AB928 + Pegylated Liposomal Doxorubicin Experimental: Dose Expansion-Ovarian-Arm 2, dose from Arm A for AB928 + Pegylated Liposomal Doxorubicin Experimental: Dose Expansion-TNBC-Arm 3, dose from Arm B for AB928 + Nanoparticle Albumin-bound Paclitaxel Experimental: Dose Expansion-TNBC-Arm 4, dose from Arm C for AB928 + IPI-549 + Pegylated Liposomal Doxorubicin	Triple Negative Breast Cancer (TNBC) Ovarian Cancer	Recruiting	NCT03719326
	AB928 mFOLFOX	A2AR/A2BR Chemotherapy	AB928: Dual A2AR and A2BR Antagonist	Phase 1	Experimental: Dose Escalation, AB928 + mFOLFOX Experimental: Dose Expansion-GE, dose from escalation for AB928 + mFOLFOX Experimental: Dose Expansion-CRC, dose from escalation for AB928 + mFOLFOX	GastroEsophageal Cancer (GE) Colorectal Cancer (CRC)	Recruiting	NCT03720678
	AB928 Zimberelimab (AB122)	A2AR/A2BR PD-1	AB928: Dual A2AR and A2BR Antagonist	Phase 1	Experimental: Dose Escalation, AB928 + fixed dose of Zimberelimab (AB122) Experimental: Dose Expansion-Renal Cell Carcinoma, recommended dose for expansion AB928 + Zimberelimab (AB122) Experimental: Dose Expansion, recommended dose for expansion AB928 + Zimberelimab (AB122)	Non-small Cell Lung Cancer Squamous Cell Carcinoma of the Head and Neck Breast Cancer Colorectal Cancer Melanoma Bladder Cancer Ovarian Cancer Endometrial Cancer Merkel Cell Carcinoma GastroEsophageal Cancer Renal Cell Carcinoma Castration-resistant Prostate Cancer	Recruiting	NCT03629756
	AB928 AB154 Zimberelimab (AB122)	A2AR/A2BR TIGIT PD-1	AB928: Dual A2AR and A2BR Antagonist	Phase 2	Experimental: Arm 1, Zimberelimab Experimental: Arm 2, AB154 + Zimberelimab Experimental: Arm 3, AB928 + AB154 + Zimberelimab	Non-Small Cell Lung Cancer Non-squamous Non-Small Cell Lung Cancer Squamous Non-Small Cell Lung Cancer Lung Cancer	Recruiting	NCT04262856
	AB928 Zimberelimab (AB122) Carboplatin Pemetrexed Pembrolizumab	A2AR/A2BR PD-1 Chemotherapy	AB928: Dual A2AR and A2BR Antagonist	Phase 1	Experimental: Dose Escalation Arm A, AB928 + Carboplatin + Pemetrexed Experimental: Dose Escalation Arm B, AB928 + Carboplatin + Pemetrexed + Pembrolizumab Experimental: Dose Expansion Arm 1, recommended dose for expansion AB928 + Carboplatin + Pemetrexed in patients harboring sensitizing EGFR mutation Experimental: Dose Expansion Arm 2, recommended dose for expansion AB928 + Carboplatin + Pemetrexed + AB122 in patients harboring sensitizing EGFR mutation	Non-Small Cell Lung Cancer Metastatic Non-Small Cell Lung Cancer Non-squamous Non-small Cell Neoplasm of Lung Sensitizing EGFR Gene Mutation	Recruiting	NCT03846310
A2BR	PBF-1129	A2BR	PBF-1129: A2BR Antagonist	Phase 1	Experimental: PBF-1129_40 mg Experimental: PBF-1129_80 mg Experimental: PBF-1129_160 mg Experimental: PBF-1129_320 mg	Non-Small Cell Lung Cancer	Recruiting	NCT03274479

Taking advantage of strong associations of CD73 with molecular and genetic alterations (e.g., *KRAS* mutation and EGFR alterations) may benefit GI cancers. Combination studies of CD73 inhibitors with anti-EGFR therapy and/or tyrosine kinase inhibitors are in clinical trials for managing resistance (Table 3). In CRC, high CD73 predicts patients benefiting from cetuximab (anti-EGFR therapy) (234). Benefits are the same for both wild-type and mutant *KRAS* tumors (234). It would be interesting to see the performance of combination cetuximab with CD73 inhibitors in preclinical CRC studies considering that inflammation is a mechanism of resistance to cetuximab (259). In melanoma, combination BRAF and MEK inhibitors with an A2AR antagonist induces significant tumor control in preclinical studies (41). MEK is a promising target for *KRAS, NRAS*, and *BRAF* mutant tumors and is being targeted in CRC (260). Recently, MEK inhibitor, cobimetinib, combined with anti-PD-L1 therapy (atezolizumab) failed to improve survival in microsatellite-stable metastatic CRC patients in a phase 3 clinical trial (261). Could the inclusion of A2AR antagonists be key to the success of these studies? AMG510, a selective inhibitor for *KRAS* (G12C) recently showed promising antitumor effects, including increasing ICI therapy sensitivity in preclinical models (262). Its combination with CD73/adenosine receptor blockade may be a promising future approach. AMG510 is in clinical trials (NCT03600883). Mentioned previously, hyperoxia induces antitumor immunity in preclinical studies, which involves the downregulation of many adenosine pathway genes (102, 108). With drug toxicity being a concern with studies pushing past two targets, approaches like this that can simultaneously dampen multiple immune checkpoints may be better tolerated and provide greater benefit (263). Although a drawback of hyperoxia therapy is that it may/does cause tissue damage (263, 264), it is interesting to consider whether this response also benefits in helping to recover antitumor immunity. Hyperoxia is in clinical trials for many conditions/diseases (ClinicalTrials.gov; hyperoxia, 87 studies).

## Conclusions

Immunotherapy in GI cancers currently benefits only a few patients. Blocking adenosine signaling by inhibiting CD73 and/or A2AR/A2BR antagonism has the potential to improve antitumor immunity in these tumors. However, identifying which patients may benefit stands in the way. To aid in these efforts, a better understanding of CD73 in human GI cancers is greatly needed. This includes initiating studies that assess CD73 in addition to other ecto-enzymes involved in extracellular adenosine synthesis and metabolism as well as their association with key molecular and genetic features. A focus of CD73 expression in primary, pretreatment, and relapsed samples will also be of great value in addition to identifying predictive biomarkers or gene signatures relating to efficacy of CD73/adenosine receptor blockade. Mechanistically, studies assessing CD73/extracellular adenosine receptor activity in humanized and autochthonous tumor mouse models and patient-derived organoids will provide needed insight into the role of CD73/extracellular adenosine in these tumors. Moreover, studies in HCC have revealed CD73 overexpression in human tumors can be misleading. Future studies also incorporating this insight have the best chance of helping to better define CD73 in GI cancers.

## Author's Note

The figure was created using Servier Medical ART templates, which are licensed under a Creative Commons Attribution 3.0 Unported License; https://smart.servier.com.

## Author Contributions

JH, LP, OV, and JB drafted, edited, and revised the manuscript. OV developed the manuscript figure.

### Conflict of Interest

The authors declare that the research was conducted in the absence of any commercial or financial relationships that could be construed as a potential conflict of interest.
